# Effects of Increasing Balance Task Difficulty on Postural Sway and Muscle Activity in Healthy Adolescents

**DOI:** 10.3389/fphys.2019.01135

**Published:** 2019-09-03

**Authors:** Arnd Gebel, Benjamin Lüder, Urs Granacher

**Affiliations:** Division of Training and Movement Sciences, Research Focus Cognition Sciences, University of Potsdam, Potsdam, Germany

**Keywords:** balance training, training intensity, youth, muscle coactivation, balance strategy

## Abstract

Evidence-based prescriptions for balance training in youth have recently been established. However, there is currently no standardized means available to assess and quantify balance task difficulty (BTD). Therefore, the objectives of this study were to examine the effects of graded BTD on postural sway, lower limb muscle activity and coactivation in adolescents. Thirteen healthy high-school students aged 16 to 17 volunteered to participate in this cross-sectional study. Testing involved participants to stand on a commercially available balance board with an adjustable pivot that allowed six levels of increasing task difficulty. Postural sway [i.e., total center of pressure (CoP) displacements] and lower limb muscle activity were recorded simultaneously during each trial. Surface electromyography (EMG) was applied in muscles encompassing the ankle (m. tibialis anterior, medial gastrocnemius, peroneus longus) and knee joint (m. vastus medialis, biceps femoris). The coactivation index (CAI) was calculated for ankle and thigh muscles. Repeated measures analyses of variance revealed a significant main effect of BTD with increasing task difficulty for postural sway (*p* < 0.001; *d* = 6.36), muscle activity (*p* < 0.001; 2.19 < *d* < 4.88), and CAI (*p* < 0.001; 1.32 < *d* < 1.41). Multiple regression analyses showed that m. tibialis anterior activity best explained overall CoP displacements with 32.5% explained variance (*p* < 0.001). The observed increases in postural sway, lower limb muscle activity, and coactivation indicate increasing postural demands while standing on the balance board. Thus, the examined board can be implemented in balance training to progressively increase BTD in healthy adolescents.

## Introduction

The development of balance with its specific components (i.e., static/dynamic steady-state, reactive, proactive balance) (Shumway-Cook and Woollacott, 2012) represents an important prerequisite for motor skill acquisition in youth (Roncesvalles et al., 2001; Mickle et al., 2011). There is evidence that balance training produces moderate-to-large effects on motor skills, balance, and sport-specific performance in youth (Malliou et al., 2004; Yaggie and Campbell, 2006; Granacher et al., 2010; Mahmoud, 2011; Boccolini et al., 2013; Walchli et al., 2017) and has the potential to reduce the risk of lower limb injuries in healthy adolescents (Malliou et al., 2004; Emery et al., 2005) and young adults (Verhagen et al., 2004). In order to optimize the effectiveness of balance training, it is crucial to elucidate the optimal combination and dosage of training modalities (e.g., training period, frequency, and volume). While training period, frequency, and volume can easily be assessed for balance training, it is more difficult to quantify balance intensity and/or balance task difficulty (BTD). This could be due to the fact that postural control is primarily neuronally and not energetically driven. In their narrative review, Taube et al. (2008) reported that adaptive mechanisms related to balance training mostly occur on a spinal (e.g., increased presynaptic inhibition) and supraspinal level (e.g., decreased corticospinal excitability). Energetically driven physical qualities like muscle strength can easily be monitored using the one repetition maximum and/or rating of perceived exertion scales (e.g., BORG, OMNI) (Robertson et al., 2003, 2004). Previously, attempts have been made to assess balance training intensity. However, either another training modality (e.g., frequency, duration) was misinterpreted as balance training intensity, or psychometric instruments (e.g., scales), only valid for other kinds of training (e.g., endurance), were used to measure intensity (Farlie et al., 2013). For instance, the BORG rating scale of aerobic exertion (Means et al., 2005) was used to quantify balance exercise intensity. Additionally, ratings of perceived exertion do not seem to be an adequate measure to quantify dosage of balance training as they measure exertion and not intensity *per se*. In fact, they were only validated for strength and endurance training but not for balance training (Robertson et al., 2003, 2004; Farlie et al., 2013).

Consequently, it is not surprising that recently published systematic reviews and meta-analyses on the effects and dose-response relationships of balance training on balance performance in youth (Gebel et al., 2018), young (Lesinski et al., 2015a) and old adults (Lesinski et al., 2015b; Farlie et al., 2018) were not able to identify a single training modality to predict balance training related effects (Farlie et al., 2018; Gebel et al., 2018). This could be due to the fact that only a limited number of training modalities (i.e., frequency, period, and volume) were included in these analyses. Consequently, Gebel et al. (2018) postulated that a measure of training intensity and/or BTD might be a promising candidate to predict balance outcomes.

The research work of Farlie et al. (2018) clearly indicated the problem, in terms of the absence of psychometrically valid tools, to quantifying balance training intensity. Therefore, it appears plausible to argue that, with balance training, intensity should be replaced by a different training modality. A promising candidate could be BTD, which can be easily modified by manipulating the base of support (BoS) and sensory inputs (proprioceptive and visual). In this context, Muehlbauer et al. (2012) showed that BTD can be increased by continuously reducing the BoS. Further, studies investigated the effects of increasing BTD on neuromuscular activity. Results showed that lower limb muscle activity (Dohm-Acker et al., 2008; Cimadoro et al., 2013) and coactivation (Donath et al., 2016) increased with increasing BTD. In fact, recent studies on the progression of BTD were mainly conducted using various environmental conditions to manipulate posture (e.g., BoS, training device, surface, vision, etc.) (Dohm-Acker et al., 2008; Muehlbauer et al., 2012; Cimadoro et al., 2013; Donath et al., 2016). However, a growing number of studies (Giboin et al., 2015, 2018; Freyler et al., 2016; Kümmel et al., 2016; Kiss et al., 2018; Makhlouf et al., 2018; Nagy et al., 2018) have clearly shown that balance is a highly task-specific. Therefore, it has to be trained and tested according to the specifics of the underlying task. Consequently, it is not possible to find answers to the question of increasing BTD using different balance tools (e.g., balance pad, board, etc.). This research question can only be answered if a single balance tool is applied that allows a gradual increase of balance task difficulty. Scientific evidence is scarce on how a graded increase of BTD using BoS only affects postural sway, lower limb muscle activity and coactivation. Based on the work of Shumway-Cook and Woollacott (1985) as well as Steindl et al. (2006), we expected that the effects of BTD on postural sway and muscle activation in adolescents were not comparable to those in adults as the processes of growth and maturation are not linear. However, to the authors’ knowledge, there are currently no studies available that examined the specific effects of a graded BTD on postural sway and neuromuscular activity in adolescents.

Therefore, the objectives of this study were to examine the effects of a gradually increasing BTD (i.e., balance board with adjustable BoS) on postural sway, lower limb muscle activity and coactivation in healthy adolescents. Based on the relevant literature, we hypothesized increases in postural sway (Muehlbauer et al., 2012; Cimadoro et al., 2013), lower limb muscle activity (Soderberg et al., 1991; Dohm-Acker et al., 2008; Cimadoro et al., 2013) and coactivation (Donath et al., 2016) with a gradually increased BTD. Moreover, we expected that the ankle muscles are mainly responsible (Dohm-Acker et al., 2008) for increases in postural sway with increasing BTD.

## Materials and Methods

### Participants

Thirteen (3 female/10 male) healthy high school students aged 16–17 years volunteered to participate in this study. Age at peak height velocity (PHV) was calculated using the sex-specific equation of Mirwald et al. (2002). The participants’ maturity level ranged from 2.3 to 4.5 years post PHV. Participants’ characteristics are summarized in Table 1. The study was approved by the local ethics committee of the University of Potsdam (application no. 18/2017). All participants and their legal guardians gave their written informed consent prior to the onset of the study. The experiment was conducted according to the latest version of the declaration of Helsinki. An *a priori* power analyses using G^∗^Power (Version 3.1.9.2, University of Kiel, Germany) (Faul et al., 2009) one group and a repeated measure ANOVA design with six measurements yielded a total sample size of *N* = 10 (effect size *f* = 0.4, α = 0.05), with an actual power of 0.91 (critical *F*-value = 2.42). Effect size was estimated using previously published work on the effects of different unstable supports on muscle activity in young adults (Cimadoro et al., 2013).

**TABLE 1:** Participants’ characteristics (mean ± standard deviation).

	**Total (*N* = 13)**	**Male (*n* = 10)**	**Female (*n* = 3)**
Age (years)	16.9 ± 0.5	17.1 ± 0.6	17.1 ± 0.4
Body height (cm)	176.4 ± 6.5	178.1 ± 5.5	170.9 ± 4.9
Body mass (kg)	67.4 ± 6.2	68.1 ± 6.0	70.2 ± 4.6
Maturity status (years after PHV)	3.0 ± 0.6	2.9 ± 0.4	3.7 ± 0.6

*PHV = peak height velocity.*

### Experimental Procedure

A single group design was used to examine the effects of increasing BTD on balance performance and leg muscle activity/coactivation in adolescents. For this purpose, participants attended the lab for one experimental session. Every session started with a standardized familiarization phase to introduce the balance task and the multi-directional balance training device (balance board). Subsequently, surface electrodes were attached to the shank and thigh muscles of the non-dominant leg. Leg dominance was assessed using the lateral preference inventory (Coren, 1993). Thereafter, participants performed three sets of six balance tasks. Each set consisted of a different randomized order of the six levels of BTD. Overall, testing of one participant comprised 18 trials with each trial lasting 30 s. To assess postural sway, center of pressure (CoP) displacements were measured using two measuring sensor mats (novel GmbH, Munich, Germany) which were placed on the balance board (Wobblesmart©, Artzt GmbH, Dornburg, Germany). Lower limb muscle activity was assessed using surface electromyography (EMG) and synchronized with CoP data. Anthropometrics were tested using a stadiometer (seca 213, seca Gmbh, Hamburg, Germany) and a bioimpedance analysis system (InBody 720, BioSpace, Seoul, South Korea).

### Balance Task

**FIGURE 1:**
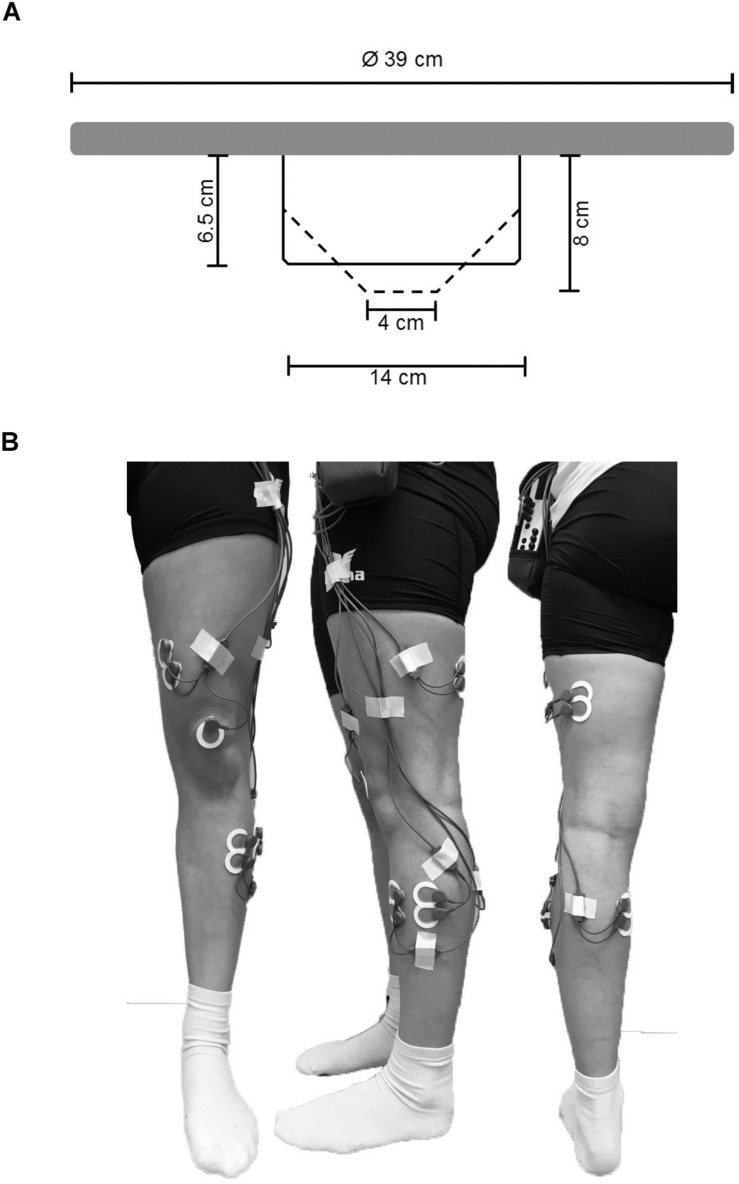
**(A)** Schematic representation of the used balance board and its mechanically adjustable pivot. By rotating clockwise the pivots diameter of the contact area is reduced (reduction in BoS) and the level of BTD increases. Solid lines represent the pivots position at BTD level 1, dashed lines represent the pivots position at BTD level 6. **(B)** Electrode sights used for respective EMG recordings of the musculus tibialis anterior (TA), m. peroneus longus (PL), m. gastrocnemius medialis (GM), m. vastus medialis (VM) and m. biceps femoris (BF) from ventral, lateral, and dorsal view.

All balance tasks were executed without shoes in bipedal upright stance on the balance board for a duration of 30 s. Every test trial started from a standardized position where participants held on to a handrail in front of them to allow quiet stance and to bring the balance board in horizontal position. During testing, participants were asked to stand in bipedal stance with knees slightly flexed at approximately 30°, to hold hands akimbo and to fixate their gaze at a cross on a nearby wall (3 m distance). During measurement, participants were instructed to keep the balance board as still as possible in horizontal plane and to avoid ground contact with the board edges. BTD was implemented into our experimental paradigm using a commercially available multi-directional balance board (Wobblesmart©, Artzt GmbH, Dornburg, Germany). The board (standing platform with a diameter of 39 cm) is equipped with a mechanically adjustable pivot to increase task difficulty. The mechanism integrated in the pivot continuously elevates the balance platform by a gradual clockwise rotation from initially 6.5 cm (level 1) to 8 cm (level 6) which simultaneously reduces the BoS diameter of the pivot from approximately 14 to 4 cm (Figure 1A).

### Measurement of Postural Sway

Postural sway in the form of total CoP displacements (combined medio-lateral and anterior-posterior direction) was assessed as a measure of performance on the balance board for 30 s (Scoppa et al., 2013) using a pressure distribution measuring system (Pedar©, novel GmbH, Munich, Germany). For this purpose, two sensor mats (Posturo S2094, novel GmbH, Munich, Germany) were placed on the balance board and fixed with double sided adhesive tape to prevent mats from slipping. The CoP displacements were recorded with 220 sensors (sensor dimensions: 20 × 20 mm) per mat (mat dimensions: 440 × 220 mm) at the maximum sampling rate (40 Hz) allowed by the system using the Posturo 32 Expert software (version 25.3.6, novel GmbH, Munich, Germany). Synchronization between CoP and EMG data was achieved using a direct link between the Pedar© and EMG system. The Pedar© system (Posturo Sync Box, novel GmbH, Munich, Germany) generated a TTL synchronization signal from onset to offset of every trial which was received and recorded by the EMG system (TeleMyo 2400R Analog Output Receiver, Noraxon©, Scottsdale, AZ, United States). Mean total CoP displacements were calculated for every participant and each level of BTD.

### Measurement of Muscle Activity

During each test trial, EMG activity of ankle [m. tibialis anterior (TA), medial gastrocnemius (GM), peroneus longus (PL)] and thigh muscles [m. vastus medialis (VM), biceps femoris (BF)] was recorded using circular bipolar surface electrodes (Ambu©, type Blue Sensor P-00-S/50, Ag/AgCl,13.2 mm, center-to-center distance 25 mm, Ballerup, Denmark). According to SENIAM guidelines (Hermens et al., 2000) and prior to the location of the electrodes on the respective muscle bellies (Figure 1B), the skin was prepared by shaving, slightly roughening, degreasing, and disinfecting to obtain an inter-electrode impedance below 5 kΩ. EMG signals were amplified, transmitted telemetrically (TeleMyo 2400 G2, Noraxon©, Scottsdale, AZ, United States), and recorded at a sampling rate of 1,500 Hz. For offline analysis, raw data were digitally band-pass filtered (10–500 Hz) followed by a moving-root-mean-square filter with a time constant of 50 ms according to the processing routine previously reported (Prieske et al., 2014, 2017) running the MyoResearch XP Master edition software (version 1.08.17, Noraxon©, Scottsdale, AZ, United States). As this cross-sectional study was carried out using a within-subject design in a single session with a fixed electrode setup, non-normalized EMG data was used for analyses (De Luca, 1997; Halaki and Gi, 2012). EMG was defined as the mean amplitude voltage in the time interval determined by the TTL-signal. First, the mean EMG amplitude was averaged across the three trials within every of the six conditions for each muscle and participant and used for analyses. Further, to analyze the effect of BTD on ankle and thigh muscle activity, the aggregated mean EMG amplitude was calculated for all ankle (TA, GM, and PL) and thigh (VM and BF) muscles. We applied this more global approach of analysis additionally because the multi-directionality of the balance task does not allow for differentiation between agonistic and antagonistic muscles. Moreover, muscle coactivation was computed for GM and TA as well as for VM and BF from the respective EMG mean amplitudes. We used the following formula according to Donath et al. (2016) to calculate the coactivation index (CAI):

$$\text{CAI}=\frac{\text{Mean EMG amplitude of the less active muscle}}{\text{Mean EMG amplitude of the more active muscle}}$$

The CAI (arbitrary values between 0 and 1) is used as an estimator of increasing joint stiffness (Donath et al., 2016) to maintain stability by a more rigid posture (Hortobágyi and DeVita, 2000; Benjuya et al., 2004).

### Statistical Analyses

All statistical tests were performed using SPSS (Version 25, IBM, Chicago, IL, United States). Behavioral (total CoP-displacements) and electrophysiological (EMG) data were tested for normal distribution using the Shapiro–Wilk test. Repeated measures analyses of variance (rmANOVA) were computed separately for postural sway (total CoP displacements), lower limb muscle activity (for individual muscles and aggregated ankle and thigh muscles), and CAI (for TA-GM and VM-BF) as dependent variables. The six levels of BTD were added as repeating factors. If significant main effects of BTD were registered, *post hoc* tests were applied using Bonferroni-corrected paired *t*-tests. Thus, it was possible to identify BTD-specific increases in postural sway (total CoP displacements), muscle activity (mean EMG amplitude for ankle and thigh muscles respective), and muscle coactivation (CAI for TA-GM and VM-BF) between single BTD levels. Where appropriate, the Greenhouse-Geisser correction for non-sphericity was applied. In addition, two forward multiple regression analyses were applied to identify which set of muscles (ankle and/or thigh muscles) and single muscles (TA, PL, GM, VM, and BF) best predict total CoP displacements. The level of significance for all statistical analyses was set at *p* ≤ 0.05. Effect estimates of partial eta-squared (η_p_^2^) were converted into Cohen’s *d* and interpreted according to Cohen (1988) with ≥0.2 as small, ≥0.5 as medium, and ≥0.8 as large effect.

## Results

### Effects of Balance Task Difficulty on Postural Sway

**FIGURE 2:**
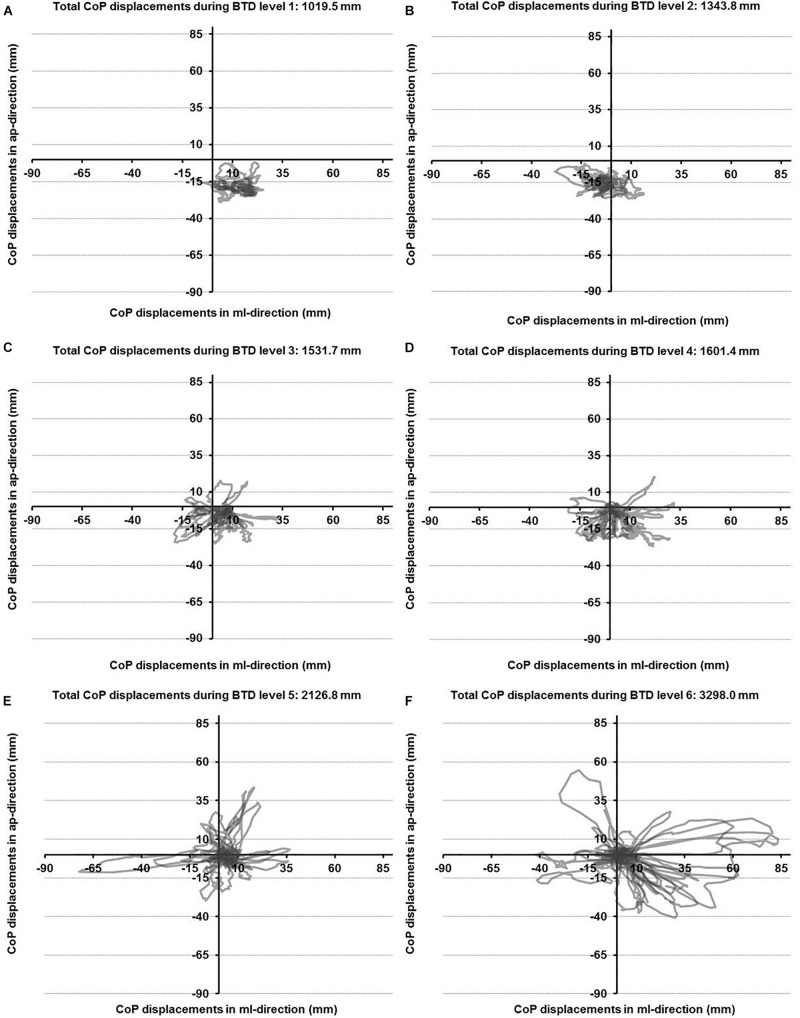
Center of pressure (CoP) displacements in anterior-posterior (ap) and medio-lateral (ml) directions for a representative participant during all six levels of balance task difficulty (BTD) for level 1 to level 6 **(A–F)**.

**FIGURE 3:**
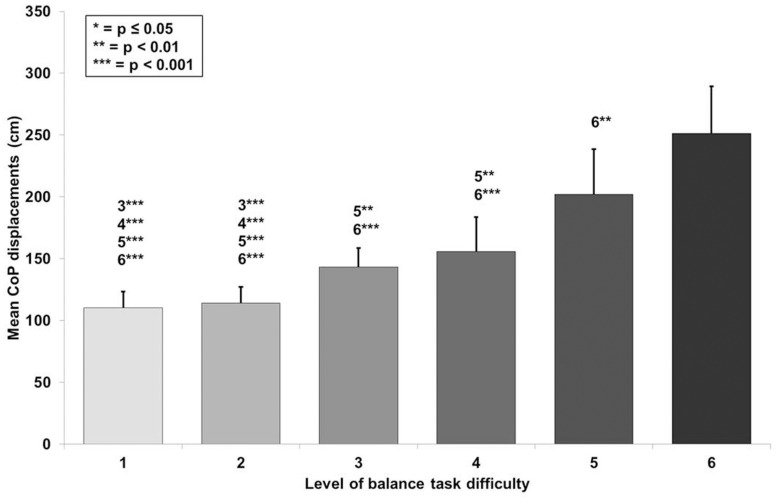
Values of the mean center of pressure (CoP) displacements with standard deviation for all six levels of balance task difficulty. Significant differences between levels are indicated by level number with respective asterisks according to the *p*-values defined in the legend.

The CoP displacements in anterior-posterior and medio-lateral direction for a representative participant across the six levels of BTD are given in Figures 2A–F. The rmANOVA revealed a large main effect of BTD (*F*_(__2__.__4__, 29__.__4__)_ = 121.6, *p* < 0.001; *d* = 6.36) for postural sway (total CoP displacements). *Post hoc* tests identified significant differences in postural sway (Figure 3) between all levels (all *p*-values ≤ 0.005, 1.52 ≤ *d* ≤ 5.91) except between level 1 and 2 as well as between level 3 and 4.

### Effects of Balance Task Difficulty on Lower Limb Muscle Activity

Statistical analyses revealed significant large-sized effects (*p* < 0.001, 2.19 ≤ *d* ≤ 4.88, Table 2) of increasing task difficulty on the individual muscles activity (i.e., TA, GM, PL, VM, BF). Adjusted *post hoc* tests showed significant differences in muscle activity between low and high levels of task difficulty (all *p*-values ≤ 0.043, Table 2).

**TABLE 2:** Mean EMG amplitudes for the individual muscles and the six levels of balance task difficulty.

	**Level of balance task difficulty (Mean ± SD)**						**RmANOVA**		***Post hoc***	
			
	**1**	**2**	**3**	**4**	**5**	**6**	***p*-value**	**Cohen’s *d***	**Difference between levels**	***p*-values**
TA (μV)	10.4 ± 10.9	12.4 ± 12.9	31.2 ± 19.9	46.2 ± 29.8	66.5 ± 38.9	69.2 ± 26.5	<0.001	3.64	1 and ≥3; 2 and ≥3; 3 and ≥4; 4 and 5	≤0.022
GM (μV)	12.9 ± 5.2	20.6 ± 13.8	47.6 ± 30.1	49.4 ± 30.3	60.6 ± 31.1	62.1 ± 32.8	<0.001	2.63	1 and ≥3; 2 and ≥5; 3 and ≥5; 4 and 6	≤0.043
PL (μV)	13.1 ± 7.8	17.6 ± 7.4	31.7 ± 10.9	39.7 ± 12.7	47.9 ± 15.8	46.8 ± 11.2	<0.001	4.88	1 and ≥2; 2 and ≥3; 3 and ≥4; 4 and ≥5	≤0.027
VM (μV)	9.5 ± 8.5	9.1 ± 7.8	17.7 ± 10.2	22.1 ± 11.5	27.5 ± 11.6	28.1 ± 11.7	<0.001	3.10	1 and ≥3; 2 and ≥3; 3 and ≥5	≤0.025
BF (μV)	12.2 ± 11.4	14.8 ± 11.3	21.7 ± 13.5	21.9 ± 8.9	26.2 ± 7.0	28.3 ± 8.3	<0.001	2.19	1 and ≥4; 2 and ≥4	≤0.015

*BF = musculus biceps femoris; GM = m. gastrocnemius medialis; PL = m. peroneus longus; TA = m. tibialis anterior; VM = m. vastus medialis.*

**FIGURE 4:**
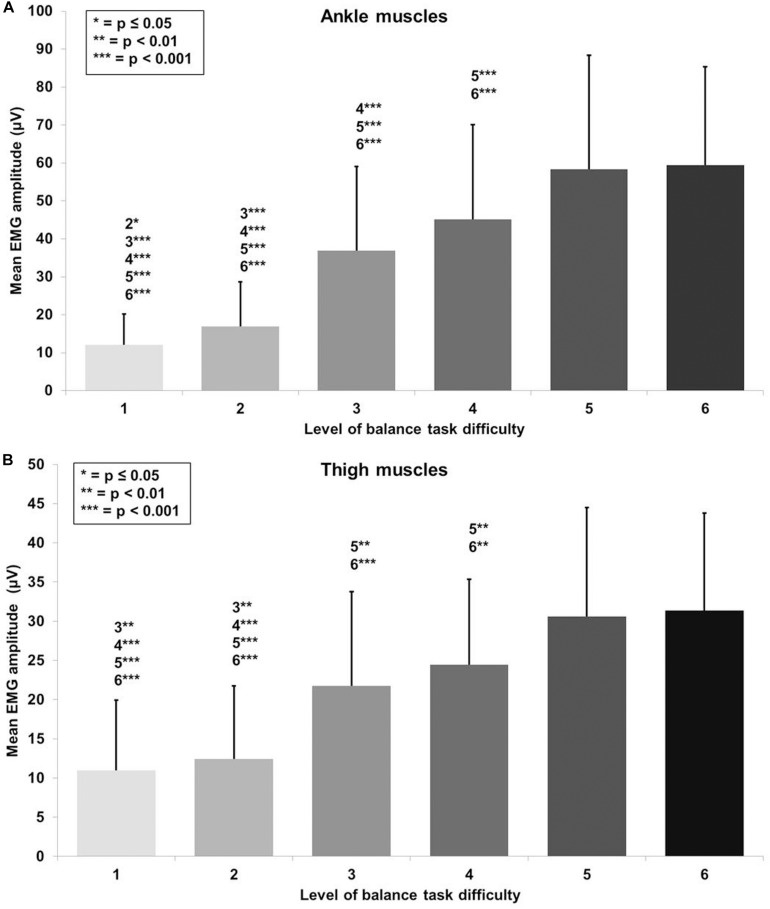
Absolute mean EMG amplitude values with standard deviation for **(A)** the ankle (tibialis anterior, peroneus longus, gastrocnemius medialis) and **(B)** thigh muscles (vastus medialis, biceps femoris) and all six levels of balance task difficulty. Significant differences between levels are indicated by level number with respective asterisks according to the *p*-values defined in the legend.

A large main effect of BTD was observed for ankle muscle activity (*F*_(__1__.__9__, 72__.__5__)_ = 81.5, *p* < 0.001, *d* = 2.93) in terms of mean EMG amplitude across the muscles (TA, GM, and PL). *Post hoc* tests with Bonferroni correction showed significant differences in muscle activity (Figure 4A) dependent on BTD between all levels (all *p*-values ≤ 0.039, 0.34 ≤ *d* ≤ 2.53) except between level 5 and 6.

Thigh muscle activity (VM, BF) showed a large main effect for BTD (*F*_(__2__.__5__, 63__.__3__)_ = 40.4, *p* < 0.001, *d* = 2.54) as well. Pairwise comparison with corrected level of significance for multiple comparison showed significant differences in thigh muscle activity (Figure 4B) dependent on BTD between all levels (all *p*-values ≤ 0.008, 0.50 ≤ *d* ≤ 1.74) except between level 1 and 2, between level 3 and 4 as well as between level 5 and 6.

### Effects of Balance Task Difficulty on Lower Limb Muscle Coactivation

**FIGURE 5:**
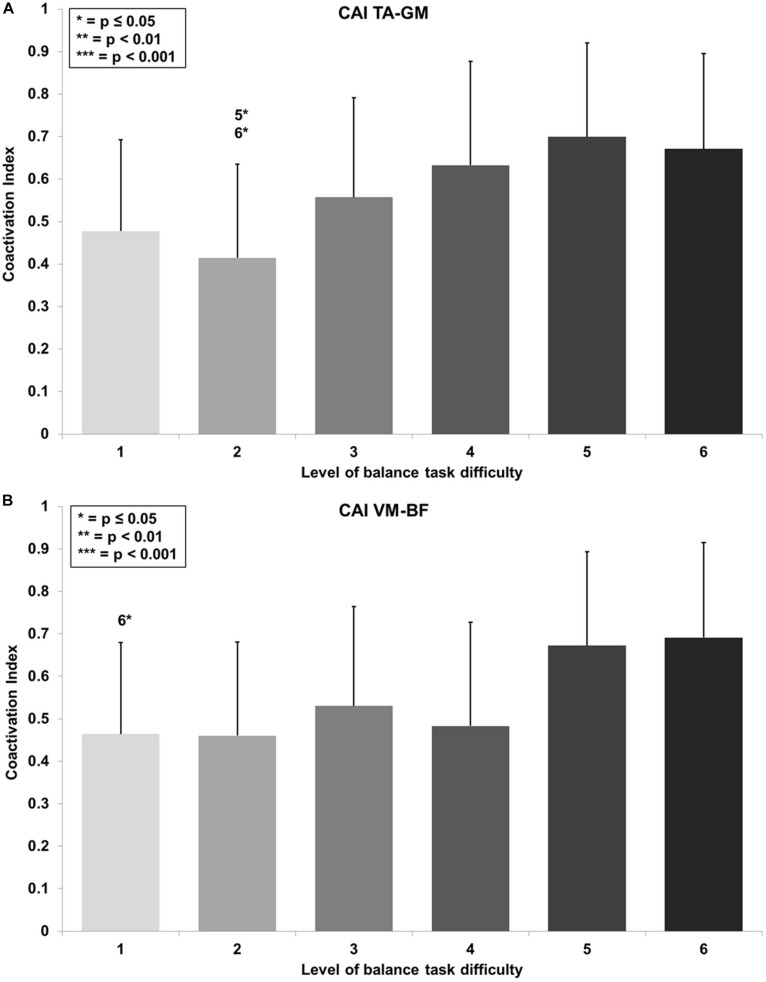
Coactivation Index (CAI) values with standard deviation for the **(A)** tibialis anterior (TA) and gastrocnemius medialis (GM) and **(B)** the vastus medialis (VM) and biceps femoris (BF) and all six levels of balance task difficulty. Significant differences between levels are indicated by level number with respective asterisks according to the *p*-values defined in the legend.

RmANOVA revealed a large main effect for BTD on lower limb muscle coactivation (*F*_(__2__.__9__, 34__.__6__)_ = 6.0, *p* = 0.002, *d* = 1.41) in terms of the CAI for muscles encompassing the ankle (TA-GM). Bonferroni-corrected pairwise comparison showed significant differences in muscle coactivation (Figure 5A) dependent of BTD between level 2 and 5 (*p* = 0.016, *d* = 1.39) as well as between level 2 and 6 (*p* = 0.022, *d* = 1.25).

Thigh CAI (VM-BF) showed a large main effect for BTD (*F*_(__2__.__9__, 35__.__0__)_ = 5.2, *p* = 0.005, *d* = 1.32) as well. *Post hoc* pairwise comparison with corrected level of significance for multiple comparison showed significant differences in muscle coactivation (Figure 5B) dependent on BTD between level 1 and 6 (*p* = 0.035, *d* = 1.02).

### Relationship Between Postural Sway and Lower Limb Muscle Activity

**FIGURE 6:**
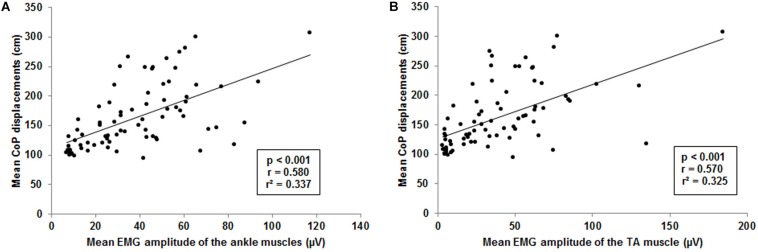
Visualization of the interrelationship between mean center of pressure (CoP) displacements and muscle activity. **(A)** Interrelation between mean CoP displacements and mean ankle muscle activity of the tibialis anterior (TA), gastrocnemius medialis and peroneus longus. **(B)** Interrelation between mean CoP displacements and mean muscle activity of the TA. Each point represents mean CoP displacements and mean muscle activity of one participant at a single level of balance task difficulty.

The forward multiple regression analysis for the muscles sets of ankle and thigh muscles revealed a single best model (*F*_(__1__, 77__)_ = 38.6, *p* < 0.001) with the ankle muscles as best predictor for the CoP displacements when level of difficulty increases (Figure 6A). All ankle muscles taken together (TA, PL, and GM) explained 33.7% of the variance (*r* = 0.580, *r*^2^ = 0.337) of the level-dependent increasing CoP displacements. Examining single muscles and muscle sets (i.e., TA and PL, TA and GM, PL and GM) encompassing the ankle, regression analysis also identified a single best model (*F*_(__1__, 77__)_ = 36.6, *p* < 0.001). The model identified the TA (Figure 6B) as best predictor explaining 32.5% of the CoP displacements variance (*r* = 0.570, *r*^2^ = 0.325). We additionally adjusted the regression analysis for potential confounders such as body height and body mass. Of note, the inclusion of these variables in our analyses did not have an impact on our findings regarding all ankle muscles (*F*_(__5__, 77__)_ = 8.2, *p* < 0.001; *r* = 0.603, *r*^2^ = 0.364) and the TA (*F*_(__5__, 77__)_ = 7.6, *p* < 0.001; *r* = 0.588, *r*^2^ = 0.346).

## Discussion

This is the first study to investigate the specific effects of a gradually increasing BTD on postural sway, lower limb muscle activity and coactivation in healthy adolescents. The main findings of this study were that an increase in the level of BTD results in an increase of postural sway and lower limb muscle activity and coactivation. Furthermore, results support the notion that at first the ankle muscles are responsible for compensating perturbations of a continuously increasing BTD.

### Effects of Balance Task Difficulty on Postural Sway

In general, the observed increase in postural sway with higher levels of BTD is consistent with findings in the literature in adults (Amiridis et al., 2003; Muehlbauer et al., 2012; Cimadoro et al., 2013). However, none of these studies examined the effects of an increasing BTD on postural sway by only reducing the BoS of a balance board while keeping the other environmental conditions constant. Muehlbauer et al. (2012), for instance, investigated CoP displacements in healthy young adults standing in four different stances (i.e., bipedal, step, tandem, unipedal) and concomitant manipulation of sensory inputs (i.e., vision, surface). The authors reported increased postural sway with reducing the BoS and sensory information. Similar findings on balance performance were reported by Donath et al. (2016) who compared postural sway of healthy young and old adults performing a single leg stance on different surfaces with open or closed eyes. The authors were able to demonstrate that an increase in task difficulty results in increasing postural sway both within and between young and old adults. When compared to our findings in adolescents, absolute CoP displacements in young adults reported by Muehlbauer et al. (2012) and Donath et al. (2016) were considerably smaller in all conditions (firm surface/eyes opened, foam surface/eyes opened, firm surface/eyes closed) of bipedal and step stance. Although the modulation of BTD was slightly different, the magnitude in performance difference suggests that the effects of BTD on postural sway in adolescents might not be comparable to those in adults. Further, in a recent study, Cimadoro et al. (2013) examined the effects of varying bases of support on postural sway in healthy young adults. Participants performed a single leg stance on three different balance boards. The authors reported higher variability of the CoP position when the balance boards’ BoS was smaller. They interpreted this variability as a decline in balance performance due to higher level of difficulty and concluded that the level of BTD could be easily increased by reducing the balance boards’ BoS. Our findings substantiate this conclusion since the systematic increase in BTD by reducing the BoS resulted in a graded increase in postural sway across all six levels. Finally, increases in postural sway with increasing BTD indicate that the used balance board seems to be well-suited to progressively increase BTD in balance training.

### Effects of Balance Task Difficulty on Lower Limb Muscle Activity

In terms of muscle activity, our results reveal an increase of the mean muscle activity across the individual muscles (i.e., TA, GM, PL, VM, and BF) as well as across the aggregated ankle (TA, PL, and GM) and thigh (VM, and BF) muscles when systematically increasing BTD. Previous research indicated that standing on unstable surfaces (i.e., wobble board, Swiss ball) results in increased lower limb muscle activity (Wahl and Behm, 2008). In this context, Borreani et al. (2014) examined the influence of different levels of stability on ankle muscle activity. Results indicated an increase in ankle muscle activity with higher levels of instability. These findings were further substantiated by Donath et al. (2016) who reported increases in relative muscle activity in individual ankle (TA, soleus, GM, PL) and thigh muscles (VM, vastus lateralis, BF, semitendinosus) during five balance tasks with varying level of task difficulty. In line with previous studies, our result support the notion that increasing BTD leads to concomitant increases in ankle and thigh muscle activation. Besides the level dependent increases of the CoP displacements, increases in ankle and thigh muscle activity might be explained by the reduced BoS at higher levels. Findings for increased ankle muscle activity are consistent with those of Soderberg et al. (1991), Dohm-Acker et al. (2008), and Cimadoro et al. (2013). These authors reported increased ankle muscle activity in the TA, GM, and PL with increasing BTD in single leg stance. This indicates the high involvement of the ankle muscles when maintaining balance under varying demands to the postural system. Additionally, thigh muscle activity also increased when the level of difficulty was increased. The level-dependent elevation of the thigh muscle activity followed a similar pattern but with smaller mean amplitude compared to those of the ankle muscles. Dohm-Acker et al. (2008) reported that EMG activity of the thigh muscles (VM and m. semimembranosus) in single leg stance remained on a steady level when BTD increased. These findings seem to be in contrast with ours. The differences could be explained by the high degree of difficulty of the balance task chosen by Dohm-Acker et al. (2008). Provided that a progressive increase in BTD is achieved by reducing the BoS, the single leg stance on an unstable surface (e.g., balance board) would be ranked on the upper end of the BTD continuum. Consequently, thigh muscle activity was increased – irrespective of the chosen unstable surface – to the point where no further increase could be observed. Taken together, the results of the present study and of Dohm-Acker et al. (2008) suggest that thigh muscle activity seems to increase until a certain level of BTD is reached and then plateaus. Further, it might be speculated that an additional increase in trunk muscle activity could have been found with increasing BTD due to changes in the postural strategy (i.e., from ankle to hip strategy) as reported by Donath et al. (2016). This assumption becomes even more apparent when looking at lower limb muscle coactivation data. Moreover, we concur with the conclusion of Dohm-Acker et al. (2008) that the thigh muscles are less involved in fine adjustments responsible to maintain or recover balance after small perturbations compared to the ankle muscles as our results also showed considerably higher activity levels. However, as the magnitude of balance perturbations increases, contributions of the thigh muscles to fine adjustments increase similarly due to a potential shift from the ankle to the hip strategy (Horak and Nashner, 1986). Ultimately, the used balance board is adequate for a continuous progression of BTD in balance training as indicated by the observed increases of lower limb muscle activity from lowest to highest level.

### Effects of Balance Task Difficulty on Lower Limb Muscle Coactivation

Coactivation of the leg muscles is influenced by a number of variables. It has been shown that with increasing age (Hortobágyi and DeVita, 2000; Benjuya et al., 2004; Donath et al., 2015; Iwamoto et al., 2017; Kurz et al., 2018) and movement velocity (Hortobagyi et al., 2009; Iwamoto et al., 2017) muscle coactivation also elevates in muscles encompassing the ankle and knee joints. In the present study, we investigated how an increase in BTD affects the coactivation of the muscles surrounding ankle and knee joints in healthy adolescents. In our study CAI values obtained for the ankle joint muscles were higher than those reported for young adults in double leg stance on unstable ground by Donath et al. (2016). These differences suggest that ankle muscle activation in adolescents and young adults is not comparable. Additionally, the CAI for muscles encompassing the knee joint showed similar values to those of young adults (Donath et al., 2016). The significant increases observed in the CAI for TA-GM and VM-BF with increasing BTD suggest that higher postural demands result in joint stiffening. Stiffening of the joints can be a mechanism to obtain more postural stability by compensating through a reduction in flexibility and mobility (Benjuya et al., 2004; Donath et al., 2016). The present findings for the ankle CAI (TA-GM) are similar to those of Iwamoto et al. (2017). The authors reported an increase of coactivation of the ankle muscles (TA and soleus) in healthy young adults when performing a balance task with higher movement velocity. Increases in coactivation of the TA and soleus were interpreted as a strategy to provide more postural stability by higher ankle joint stiffness. Further, our results indicate that the CAI (TA-GM) increase from low (level 2) to high levels (level 5 and 6) of BTD. The progressive increase of the CAI seems to be graded, even though our analyses did not yield statistical evidence for this assumption. However, the CAI for muscles encompassing the knee (VM-BF) was especially elevated for high levels (levels 5 and 6) and reached statistical significance at level 6 of the balance task. As the CAI can be used as an indicator for joint stiffening, our data suggest that low-to-medium levels of task difficulty can be compensated for using the ankle strategy. When BTD further increases, increased levels of CAI are needed to stiffen lower limb joints in order to maintain postural stability. These findings indicate that a shift from the ankle to the hip strategy occurred with increasing levels of BTD (Donath et al., 2015). In this context, recent studies (Papegaaij et al., 2016; Watanabe et al., 2018a, b) demonstrated that an increase in postural challenge resulted not only in a change of postural strategy but also in an increase of cortical control. For example, Papegaaij et al. (2016) found decreases of soleus EMG suppression induced by transcranial magnet stimulation only in a balance task with high postural challenge. Therefore, the authors assumed that these changes were related to modulation in intracortical circuits indicating increased cortical control with higher postural demands. Further, Watanabe et al. (2018a) reported that coherence in the delta-band between bilateral homologs muscles (e.g., GM-GM) and in the beta-band between unilateral muscles (GM-soleus) changed with increasing postural challenge in young but not old adults. Their results indicate a shift from bilateral synchronous to unilateral cortical control of the ankle muscles as unilateral cortical control increases when postural demands increase. Further, they assumed that this modulatory ability is impaired with increasing age as there were no changes in older adults. When related to the results of the present study, these findings suggest a shift from subcortical to cortical control processes with increasing level of BTD which might also result in increased cortical activity. In conclusion, the increase of the CAIs and the assumed change of postural strategy from ankle to hip strategy indicate higher demands to the postural control system which may also result in changes of cortical control and activity. Thus, increases in CAI values are another indicator that BTD can be progressively increased in balance training by the tested balance board.

### Relationship Between Postural Sway and Lower Limb Muscle Activity

Results of the regression analyses suggest that for the tested balance board and levels of task difficulty the strongest contributions on the muscular level for maintaining postural control were made by the ankle muscles and especially by the TA. These findings support the notion that muscle activity and postural sway are interrelated (Gatev et al., 1999; Watanabe et al., 2018a, b). Tilting movements mainly performed in the anterior/posterior (AP) direction to recover balance (Cimadoro et al., 2013) might explain TA activity as main contributor to CoP displacements although the used balance board was a multi-directional board. Additionally, due to the anatomy of the foot, the leverage in AP direction is larger and enables a more controlled force transduction to the balance board making it easier to maintain and recover the balance board in a horizontal position. Therefore, the relationship found in the present study might not only rely on compensatory mechanisms to increase stability (i.e., stiffening of the ankle joints by increasing co-activation) but also on voluntary contractions to actively control the tilt of the balance board. However, the TA may not only be responsible for dorsi-extension of the ankle but may also be involved in compensatory movements in medio-lateral direction. Activity of the TA may therefore be much more prominent than the PL and GM even if the AP direction is the preferred one to control a multidirectional balance board in bipedal stance. Nevertheless, contributions of the TA to compensatory medio-lateral movements might be limited to bipedal stance. In this context, Watanabe et al. (2018a) analyzed the relationship between CoP sway and EMG activity of the GM, gastrocnemius lateralis, and soleus in bipedal as well as unipedal stance and found that these muscles are only involved in medio-lateral sway during unipedal stance. However, the authors did not include they TA in their analyses. Therefore, assumptions on basis of the present results on the contributions of the TA to compensatory medio-lateral movements remain speculative.

### Limitations

Few potential limitations of this study warrant discussion. First, additional recordings of kinematic data (e.g., knee angle) and trunk muscle activity might have provided clearer evidence for occurred changes in the postural strategy. Further, this might have helped to elucidate how the more proximal muscles of the trunk (e.g., m. multifidus lumborum and m. internal oblique) are affected by higher levels of task difficulty (Donath et al., 2016). Therefore, our discussion on that subject remains speculative. Moreover, future studies should include the soleus muscle to examine the plantarflexor function irrespective of knee joint motion. In addition, recent studies (Papegaaij et al., 2016; Watanabe et al., 2018a, b) demonstrated increases in the cortical control of posture with increasing postural demands. It is hypothesized that increased BTD might be reflected in a shift from subcortical to cortical control processes to maintain balance. Thus, future studies need to elucidate cortical activity during the performance of balance tasks with increasing difficulty using, for instance, electroencephalography. As the experiment was part of a larger experimental setup, the additional application of kinematics and electrode locations on the trunk would have been too strenuous for the participants. Since we examined adolescents, we tried to keep the preparation phase as short as possible in order to keep the participants as focused and motivated as possible. Finally, future studies should investigate how sex and parameters like the Body Mass Index moderate balance performance with increasing task difficulty.

## Conclusion

It has previously been shown in healthy adults that the manipulation of the BoS and sensory inputs induces increasing postural demands and muscle activity. The present study is the first to examine the effects of a continuous increase in BTD on postural sway, lower limb muscle activity and coactivation in healthy adolescents. In summary, our findings revealed increased postural sway, muscle activity and coactivation with a continuous increase in BTD in healthy adolescents. Further, our results indicate an interrelationship between postural sway and lower limb muscle activity with increasing postural demands. It can be suggested that compensatory mechanisms which regulate and maintain postural stability are mainly located at the ankle but may shift to the hip with increasing level of BTD. Moreover, the findings provide evidence that the tested balance board can be used to gradually increase BTD in balance training. These insights might be helpful to optimize individual balance training regimes in the fields of rehabilitation and athletic development. While the difficulty-dependent effects on balance performance and neuromuscular activity were demonstrated, it remains unclear how increasing postural demands affect brain activity. Hence, future studies should investigate the effects of gradually increasing BTD on cortical activity in healthy adolescents.

## Ethics Statement

The study was approved by the local ethics committee of the University of Potsdam (application no. 18/2017). All participants and their legal guardians gave their written informed consent prior to the onset of the study. The experiment was conducted according to the latest version of the Declaration of Helsinki.

## Author Contributions

AG and UG conceived and designed the research. AG conducted the experiment and analyzed the data. All authors contributed to the writing of this manuscript, read, and approved the manuscript.

## Conflict of Interest Statement

The authors declare that the research was conducted in the absence of any commercial or financial relationships that could be construed as a potential conflict of interest.
